# Supplementary material for: Assortative Mating: Encounter-Network Topology and the Evolution of Attractiveness
Source: Sci Rep. 2017 Mar 27;7:45107. doi: 10.1038/srep45107 (PMC5366857; doi:10.1038/srep45107)
Supplement: Supplementary Information [file srep45107-s1.pdf]

Assortative Mating: Encounter-Network Topology and the Evolution of Attractiveness - **Supplementary Information**

S. Dipple[1,2]*, T. Jia[3], T. Caraco[4], G. Korniss[1,2], B. K. Szymanski[2,5,6]

1 Department of Physics, Applied Physics, and Astronomy, Rensselaer Polytechnic Institute, 110 8th Street, Troy, NY, 12180-3590 USA
2 Network Science and Technology Center, Rensselaer Polytechnic Institute, 110 8th Street, Troy, NY, 12180-3590 USA
3 College of Computer and Information Science, Southwest University, Chongqing, 400715, P. R. China
4 Department of Biological Sciences, University at Albany, Albany NY 12222, USA
5 Department of Computer Science, Rensselaer Polytechnic Institute, 110 8th Street, Troy, NY, 12180-3590 USA
6 Wroclaw University of Science and Technology, 50-370 Wroclaw, Poland

Transformation of the Distribution of Link Weights

We take $w_{i,j}^{\beta}$ as a realization of the continuous random variable Y, where $Y = (X * X')^{\beta}$ and X and X' are independent, identically and uniformly distributed, random variables on [0,1]. Then $Z = X * X'$ is a product of random variables [24] with the probability density $f_Z(z)$ defined as:

$$f_Z(z) = \int_{-\infty}^{\infty} f_X(x) f_{X'}(z/x) \frac{1}{|x|} dx \tag{S1}$$

where $f_X(x)$ is unity for $x \in [0,1]$. $f_{X'}(z/x)$ does not share these same bounds; rather it has range from $z/x = 0$ to $z/x = 1$. For $z \neq 0$, $f_{X'}(z/x)$ is unity for $x \in [z, \infty]$. Because $z \in (0,1]$, these bounds overlap on the interval $[z,1]$. Substituting these probability densities over the appropriate bounds yields the following.

$$f_Z(z) = \int_z^1 \frac{1}{x} dx \tag{S2}$$

$$f_Z(z) = -ln(z), z \in (0,1] \tag{S3}$$

We then change variables to $Y = Z^{\beta}$. Since probability over a differential area must be invariant under change of variable, we have:

$$|f_Y(y)dy| = |f_Z(z)dz| \tag{S4}$$

Both of these functions are positive which removes the need for absolute values. Using $z = y^{\frac{1}{\beta}}$ and $ln(z) = \frac{1}{\beta} ln(y)$, we substitute and isolate $f_Y(y)$.

*E-mail: dippls@rpi.edu

$$\frac{dz}{dy} = \frac{y^{\frac{1}{\beta}-1}}{\beta} \tag{S5}$$

$$f_Y(y) = f_Z(z)\frac{y^{\frac{1}{\beta}-1}}{\beta} \tag{S6}$$

$$f_Y(y) = -\frac{ln(y)}{\beta^2}y^{\frac{1}{\beta}-1} \tag{S7}$$

This shows that the selectivity mechanism is mathematically equivilent to having link weights with the above probability distribution.

Supplementary Figures

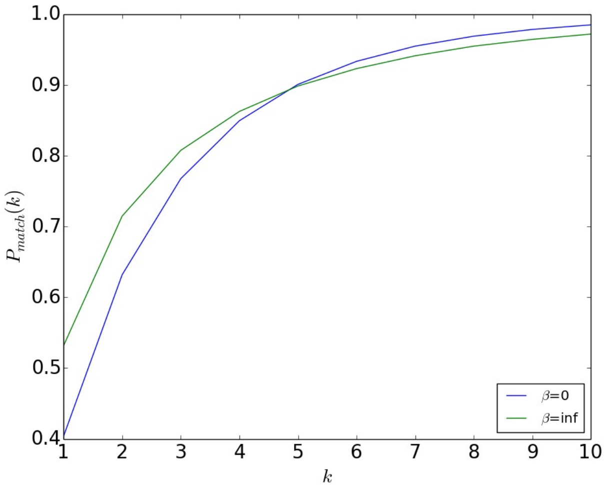

Figure S1: **Matching Probability.** 1000 realizations are produced with $\langle k \rangle = 5$. Each node is examined before and after the matching process to determine the probability that a node with a given degree will be matched.

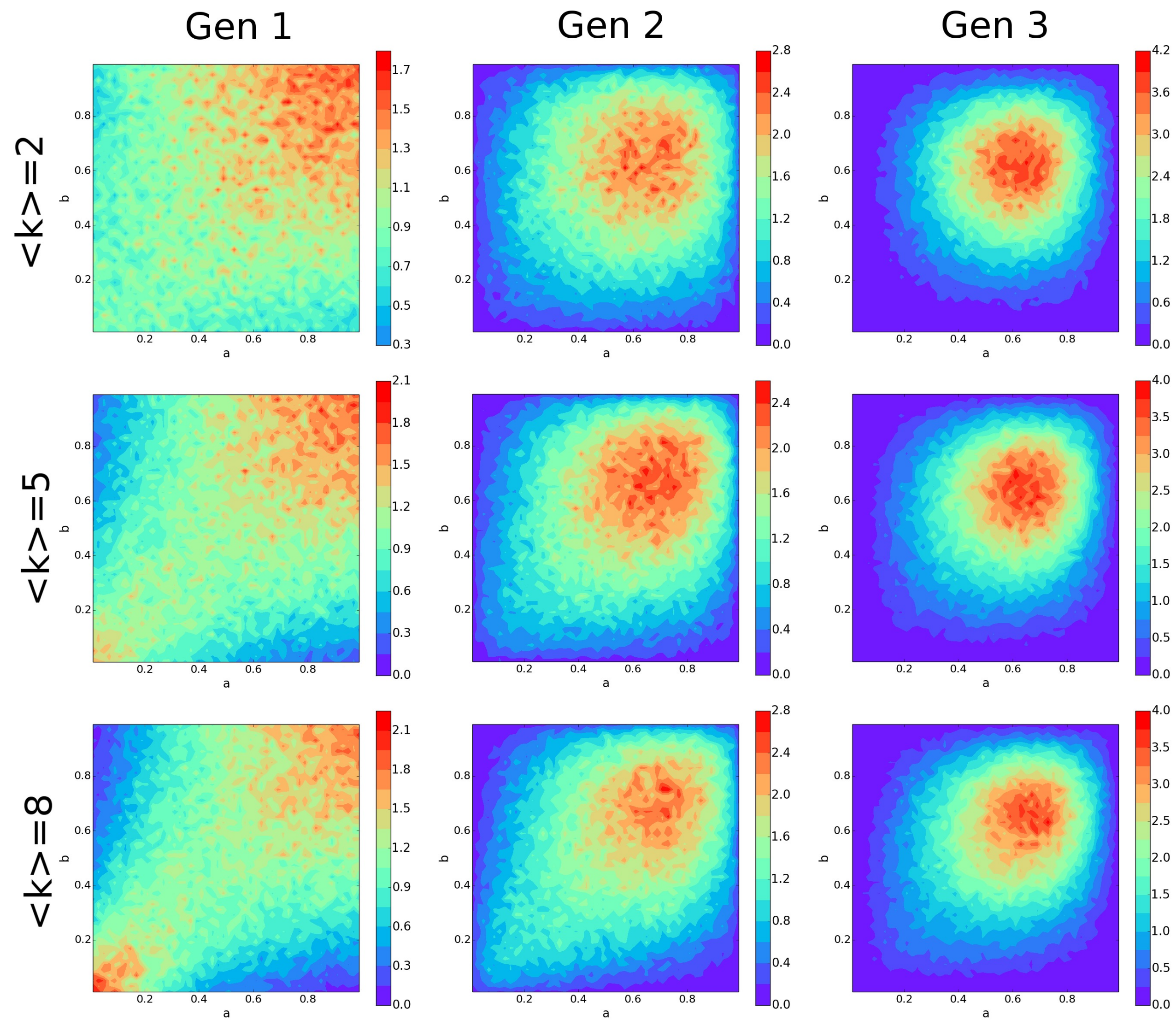

Figure S2: **Attractiveness Joint-Probability Distribution Evolution I.** Each mated pair is binned into a 0.02 by 0.02 bin according to the pair's attractiveness set. Each distribution is normalized and then averaged, and the bins are smoothed for visual purposes. The top row corresponds to $\langle k \rangle = 2$, middle row $\langle k \rangle = 5$, and bottom row $\langle k \rangle = 8$. The left column corresponds to generation zero, middle column generation one, and right column generation two. All distributions are generated with $\beta = 1$ and $G = 0.75$.

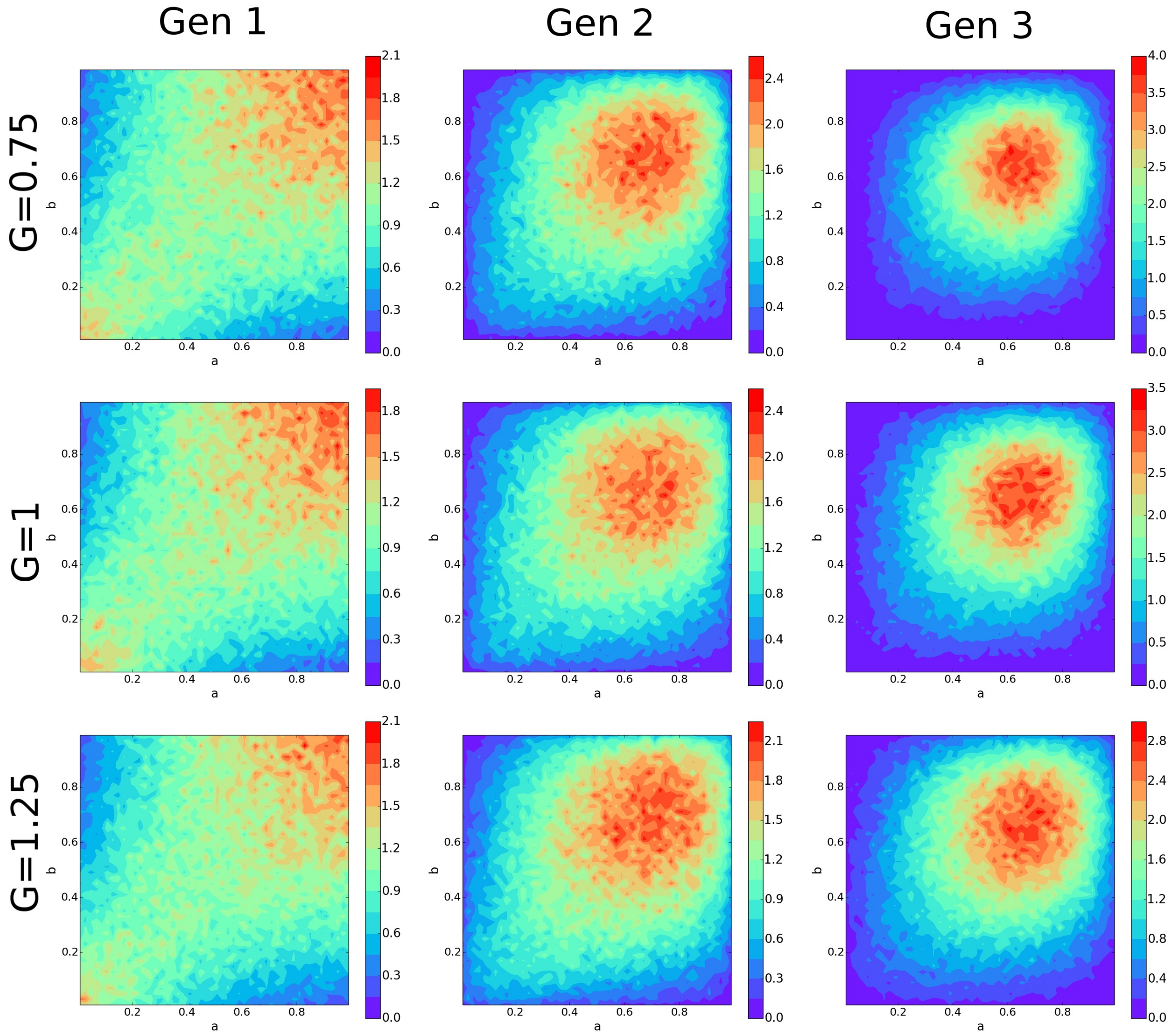

Figure S3: **Attractiveness Joint-Probability Distribution Evolution II.** Each mated pair is binned into a 0.02 by 0.02 bin according to the pair's attractiveness set. Each distribution is normalized and then averaged, and the bins are smoothed for visual purposes. The top row corresponds to $G = 0.75$, middle row $G = 1$, and bottom row $G = 1.25$. The left column corresponds to generation zero, middle column generation one, and right column generation two. All distributions are generated with $\langle k \rangle = 5$ and $\beta = 1$. All distributions in the left column are similar, as the first generation distribution is not dependent on the offspring variance.

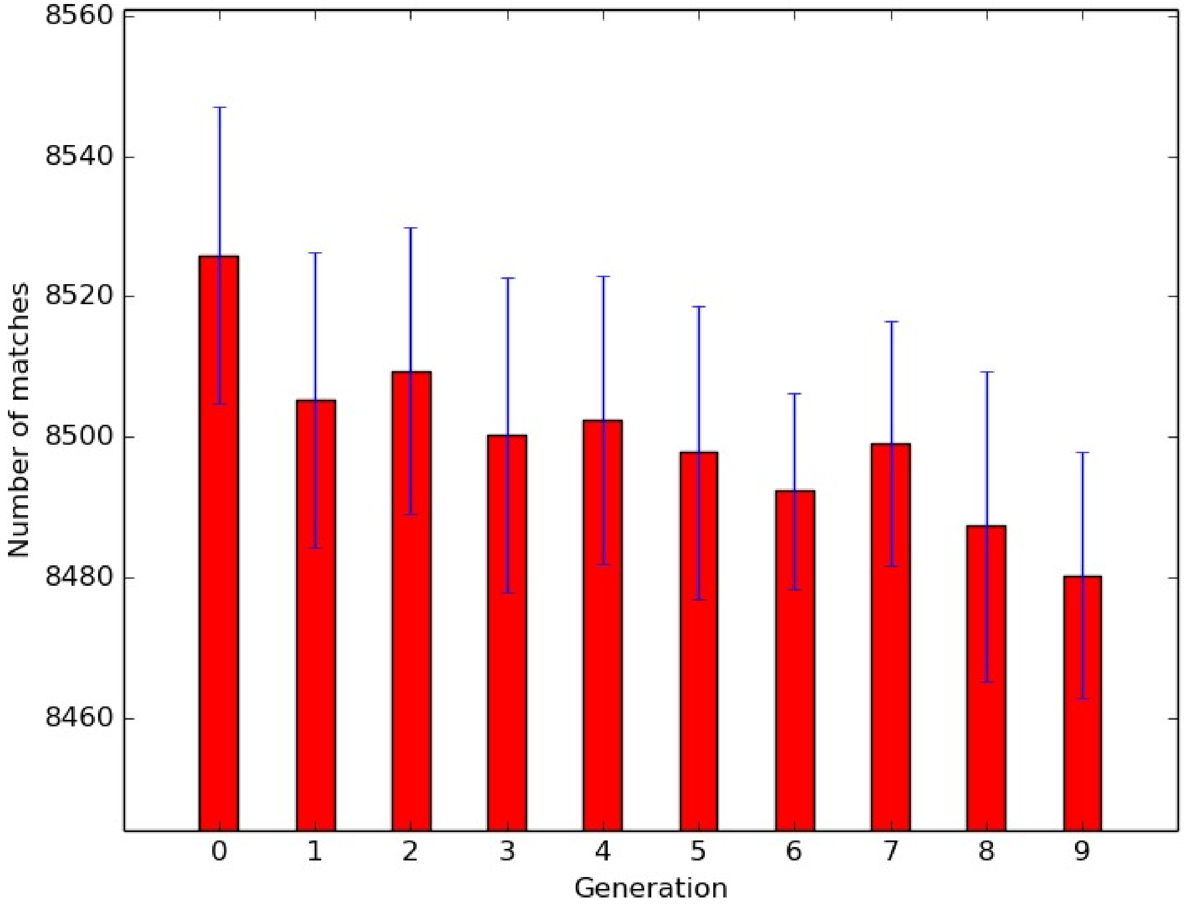

Figure S4: **Evolution of the Number of Matches.** Number of matches as a function of generation number. Twenty realizations were run with $\langle k \rangle = 5$, $\beta = 0.5$, and $G = 1.25$

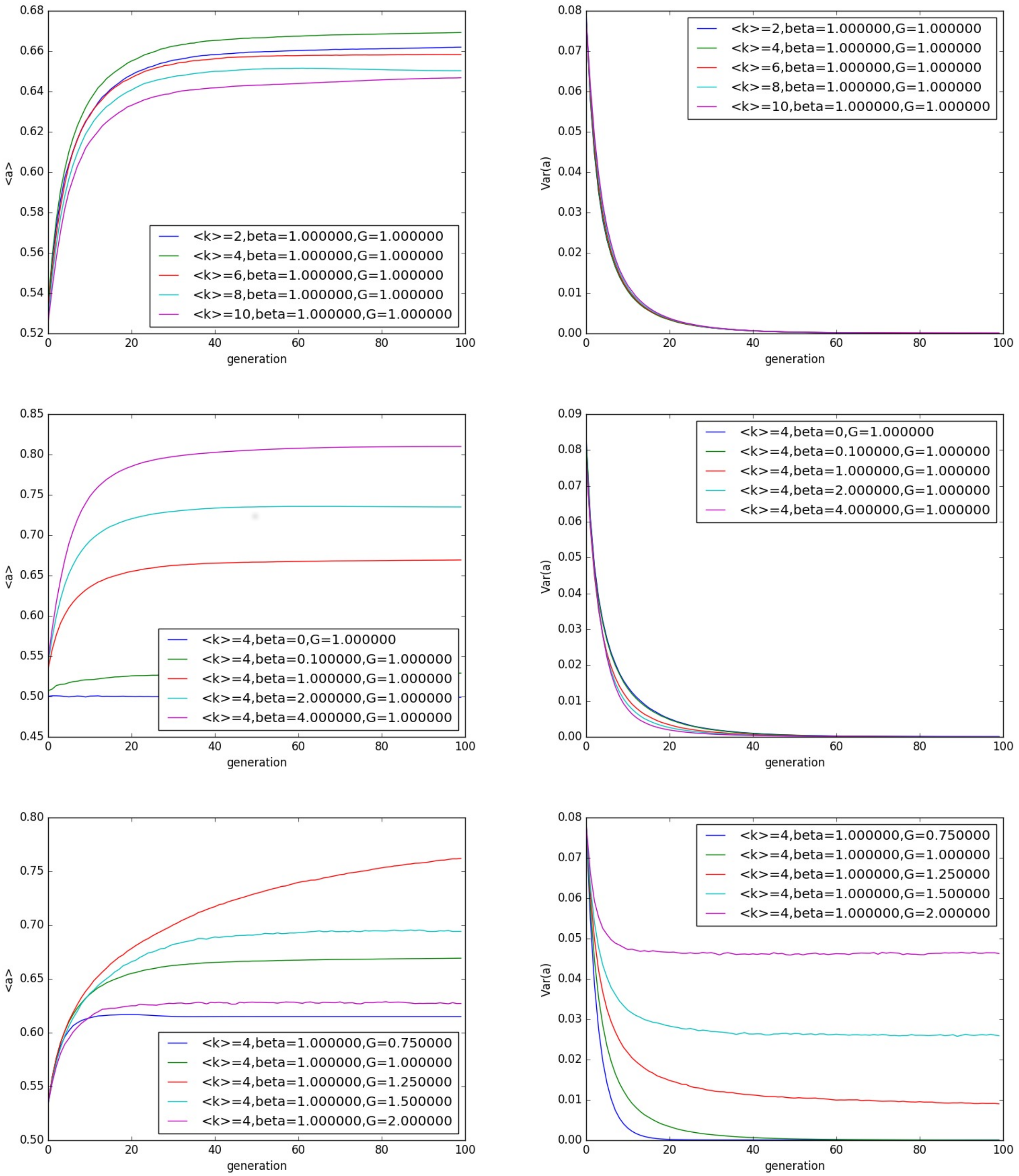

Figure S5: **Long-Term Attractiveness Behavior.** The mean $< a >$ and variance $Var(a)$ of attractiveness of group $A^{(g)}$ are calculated to 100 generations. We plot values only for $A^{(g)}$ because $A^{(g)}$ and $B^{(g)}$ are statistically equal. The means are all monotonic with most reaching a stable value within the first ten to twenty generations. The variances are all decreasing in value and only stabilizes above zero at or above a certain threshold of G slightly below $G = 1.25$. Also of interest is that $G = 1.25$ produces the largest mean at later generations for the tested sample.
